# Identification of 5 hub genes for diagnosis of coronary artery disease

**DOI:** 10.3389/fcvm.2023.1086127

**Published:** 2023-07-05

**Authors:** Pengyuan Zhu, Haitao Huang, Tian Xie, Huoqi Liang, Xing Li, Xingyi Li, Hao Dong, Xiaoqiang Yu, Chunqiu Xia, Chongjun Zhong, Zhibing Ming

**Affiliations:** ^1^ Department of Thoracic and Cardiovascular Surgery, School of Medicine, The Second Affiliated Hospital of Nantong University, Nantong University, Nantong, China; ^2^ Department of Thoracic and Cardiovascular Surgery, Tongji Hospital, School of Medicine, Tongji University, Shanghai, China; ^3^ Department of Vascular Surgery, The Second Affiliated Hospital of Nantong University, Nantong, China

**Keywords:** gene, diagnosis, CAD development, cardiovascular disease, coronary artery disease

## Abstract

**Background:**

Coronary artery disease (CAD) is a main cause leading to increasing mortality of cardiovascular disease (CVD) worldwide. We aimed to discover marker genes and develop a diagnostic model for CAD.

**Methods:**

CAD-related target genes were searched from DisGeNET. Count expression data and clinical information were screened from the GSE202626 dataset. edgeR package identified differentially expressed genes (DEGs). Using online STRING tool and Cytoscape, protein-protein reactions (PPI) were predicted. WebGestaltR package was employed to functional enrichment analysis. We used Metascape to conduct module-based network analysis. VarElect algorithm provided genes-phenotype correlation analysis. Immune infiltration was assessed by ESTIMATE package and ssGSEA analysis. mRNAsi was determined by one class logistic regression (OCLR). A diagnostic model was constructed by SVM algorithm.

**Results:**

162 target genes were screened by intersection 1,714 DEGs and 1,708 CAD related target genes. 137 target genes of the 162 target genes were obtained using PPI analysis, in which those targets were enriched in inflammatory cytokine pathways, such as chemokine signaling pathway, and IL-17 signaling pathway. From the above 137 target genes, four functional modules (MCODE1-4) were extracted. From the 162 potential targets, CAD phenotype were directly and indirectly associated with 161 genes and 22 genes, respectively. Finally, 5 hub genes (CCL2, PTGS2, NLRP3, VEGFA, LTA) were screened by intersections with the top 20, directly and indirectly, and genes in MCODE1. PTGS2, NLRP3 and VEGFA were positively, while LTA was negatively correlated with immune cells scores. PTGS2, NLRP3 and VEGFA were negatively, while LTA was positively correlated with mRNAsi. A diagnostic model was successfully established, evidenced by 92.59% sensitivity and AUC was 0.9230 in the GSE202625 dataset and 94.11% sensitivity and AUC was 0.9706 in GSE120774 dataset.

**Conclusion:**

In this work, we identified 5 hub genes, which may be associated with CAD development.

## Introduction

Coronary heart disease (CAD), as a kind of cardiovascular disease, has high incidence, mortality, recurrence rate, showing an unfavorable prognosis (1). The basic pathological change of CAD is atherosclerosis. Previous studies (2, 3) have confirmed that lipid metabolism and inflammatory response are involved in the pathogenesis of CAD. The use of antiplatelet drugs and statins has a positive effect on the prevention of CAD induced adverse events (4), However, the bleeding risk of antiplatelet therapy and the liver toxicity of statins also affect the treatment effect of CAD to a certain extent (5). Cardiac MRI, single-photon emission computed tomography and angiography are often used in the diagnosis of CAD with high accuracy, however, unstable image quality and invasive examination methods limit their clinical application. Therefore, finding a less invasive and more accurate examination method has become the focus of clinical attention (6).

With the maturation of gene sequencing technology and the reduction of cost, the application of genomics in the field of medicine has gradually expanded. Compared with the validation of a single target or pathway in traditional research ideas, bioinformatics methods based on genomics can obtain and analyze massive gene expression data in a short time, and dig into the underlying mechanisms and core pathways of diseases (7). The Gene Expression Database (GEO) of the National Center for Biotechnology Information (NCBI) is the largest disease database available (8). In this study, bioinformatics methods were used to mine CAD related gene datasets in GEO, screen DEGs and conducted functional enrichment analysis for exploring the potential targets and mechanisms of CAD, so as to improve the theoretical basis for CAD diagnosis and treatment.

## Materials and methods

### Identification of DEGs

Count expression data and clinical information of dataset GSE202625 were obtained from the public database of GEO. DEGs between CAD and the control group in the GSE202625 dataset were analyzed by edgeR package, with *p* < 0.05 and | a Fold Change (FC) | >1. 2 as a threshold for screening.

### Collection of CAD related genes

DisGeNET (http://www.disgenet.org/) was used for acquiring CAD-related targets through searching the term “coronary artery disease” on the platform. Finally, 1,708 genes were obtained.

### Functional enrichment analysis

The R software package WebGestaltR V 0.4.4 was used during GO functional enrichment analysis and KEGG pathways, with the enriched GO entries and pathways being defined as having a *p* value <0.0 5.

### PPI network construction

The intersection of GSE202625 differential genes and CAD-related targets were seen as possible therapeutic targets of CAD. String database (https://stringdb.org/, version 1 1.5) could be used for analyzing known and predicted PPI. Cytoscape (http://cytoscape.org/, version 7 2) can visualize the complex relationships.

### Gene phenotype correlation analysis

The VarElect tool is a free web-based phenotype-dependent variant/gene prioritizer. VarElect employs powerful search and scoring functions of GeneCards, an integrated genomic database, and scoring functions, and its algorithm provides inferred direct as well as indirect links between uploaded genes and inputted disease/phenotype. Therefore, the online tool affords a robust facility for ranking genes and pointing out their likelihood to be related to specific diseases. The VarElect tool (http://ve.genecards.org) assessed the association between potential therapeutic targets of CAD and “coronary artery disease” phenotype.

### Correlation between hub genes and immunity

The ESTIMATE R package, which estimates stromal and immune cells in CAD tissue based on gene expression data, which generated three scores, including (i) ImmuneScore representing the infiltration of immune cells in CAD tissue, (ii) StromalScore capturing the presence of stroma in CAD tissue, and (iii) ESTIMATEScore inferring tumor purity, were used to predict CAD purity and the presence of infiltrating stromal and immune cells in CAD tissue. And the ESTIMATE software package calculates the three scores (StromalScore, ImmuneScore, ESTIMATEScore) of the dataset GSE202625. To quantify the relative percentage of immune cells in CAD samples, 28 types of immune cells were identified with high sensitivity and specificity using the ssGSEA algorithm. Then we calculated the Pearson correlation coefficient between the immune score and hub genes.

### The calculation of mRNAsi

The expression data of pluripotent stem cell samples (ESC and iPSC) from the Progenitor Cell Biology Consortium (PCBC) database were used for predicting and calculating the stem cell index using the one class logistic regression (OCLR) method. Firstly, only the sample data of ESC and iPSC are kept, which are collectively referred to as SC samples. The Ensembl IDs of SC samples are converted into Gene Symbol and only the genes encoding proteins are kept. There are a total of 78 SC samples, expression profiles of 8,087 mRNA genes in each sample. For the obtained expression profile, the average value was used to centralize each sample. Finally, the OCLR method in R package gelnet v1.2.1 was used to calculate the weight vector of each gene for the processed data.

### Construction of diagnostic models

The SVM algorithm (9) was used to construct the diagnostic model on the GSE202625 dataset based on hub genes, and 10-fold cross-validation was performed (10). GSE120774 dataset acted as an independent validation dataset.

## Results

### Identification of target genes for CAD

**Figure 1:**
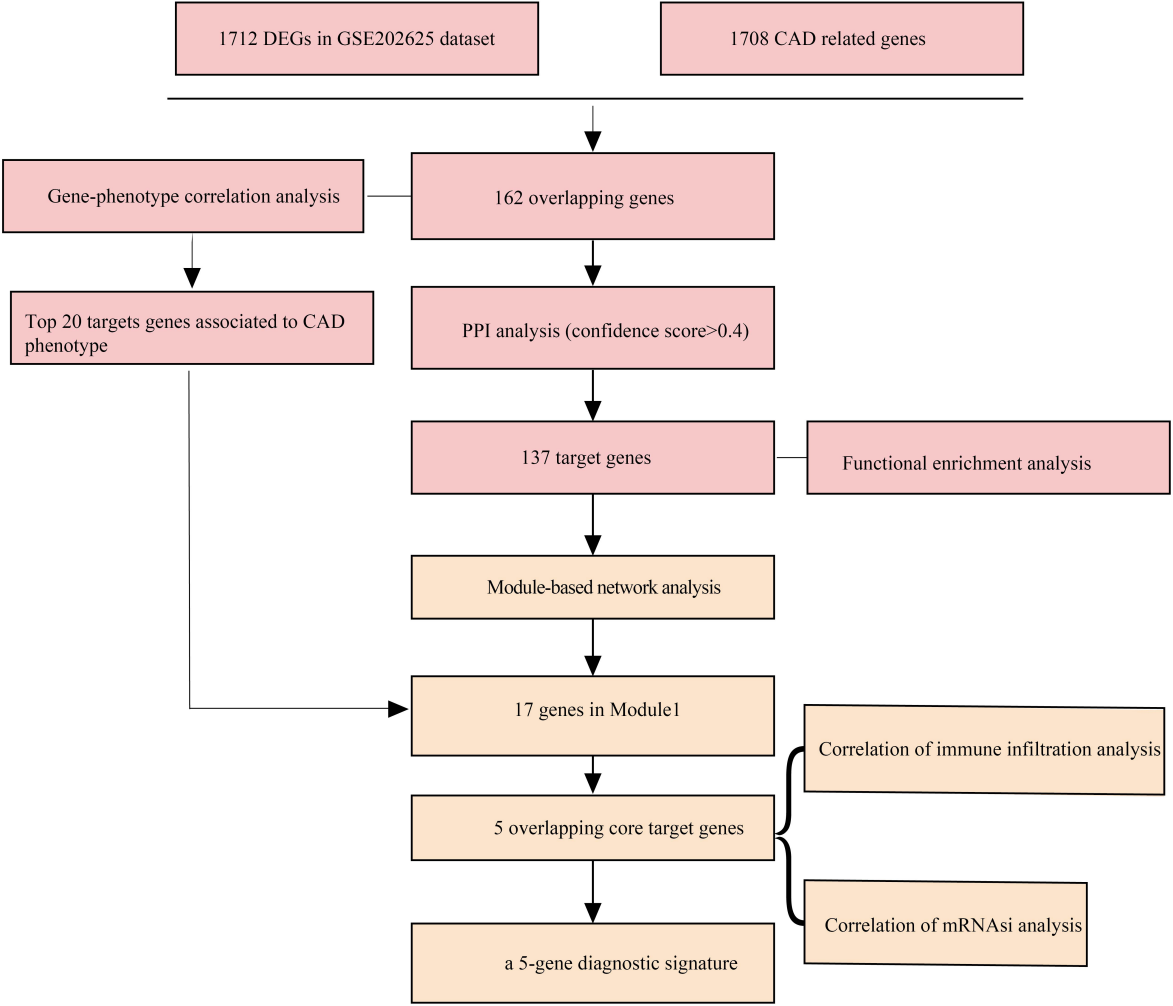
The flowchart of this study.

**Figure 2:**
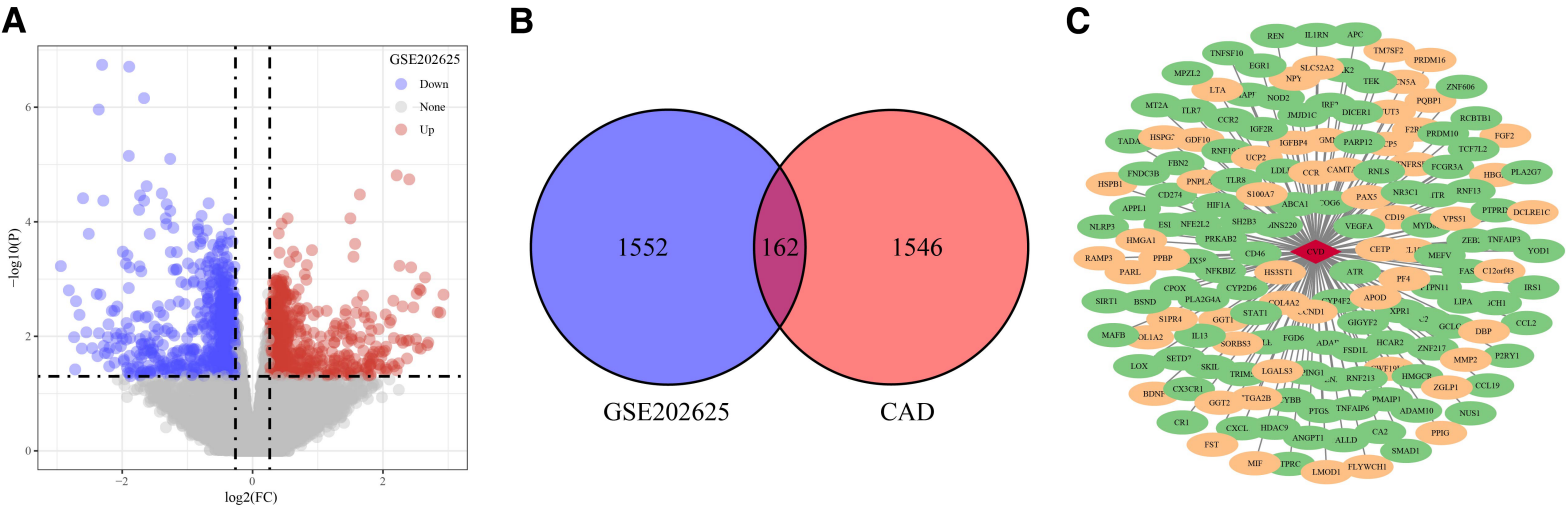
Identification of target genes. (**A**) DEGs between CAD and normal samples in GSE202625 dataset. (**B**) Venn of DEGs and CAD related genes. (**C**) Network of DEGs and CAD related genes. The red diamond in the middle is CAD, the oval is gene, the green represents down-regulated expression, and the yellow represents up-regulated expression.

The flowchart was shown in Figure 1. In the GSE202625 dataset, we identified 1,714 DEGs between CAD and normal samples (Figure 2A). By intersecting 1,708 CAD related genes and 1,714 DEGs, we screened 162 target genes (Figure 2B). We also drew a network of DEGs and CAD related genes in CAD (Figure 2C).

**Figure 3:**
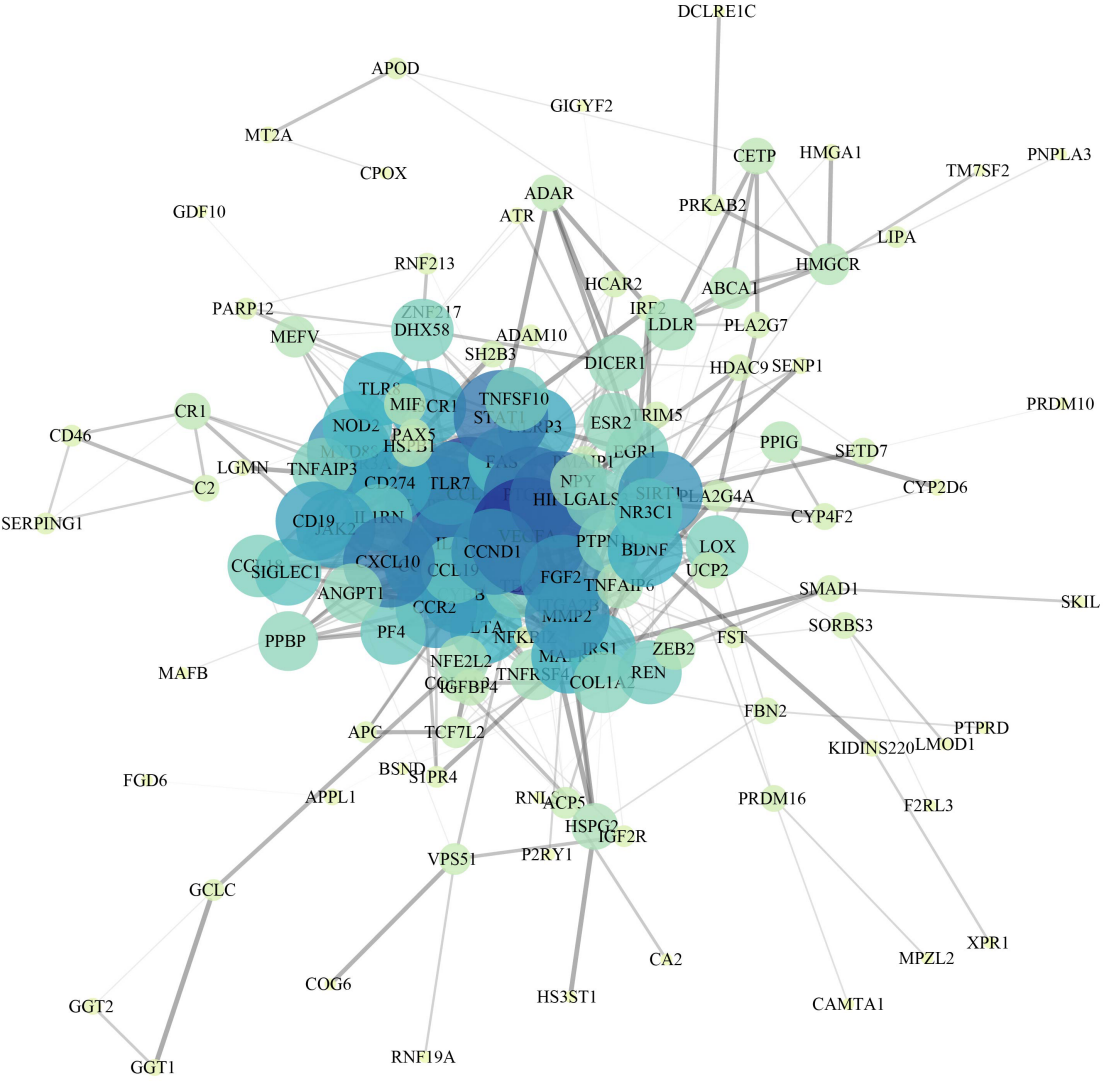
PPI network of CAD.

A total of 162 targets reserved with a confidence score > 0.4 were screened out of all the intersection targets according to the confidence range of defining PPI in String database. With the PPI from String database, to establish relationship network for the targets, Cytoscape software was used. In the PPI network, a sum of 137 target genes were kept after removing nodes with few edges (Figure 3).

### Functional enrichment analysis of 137 target genes

**Figure 4:**
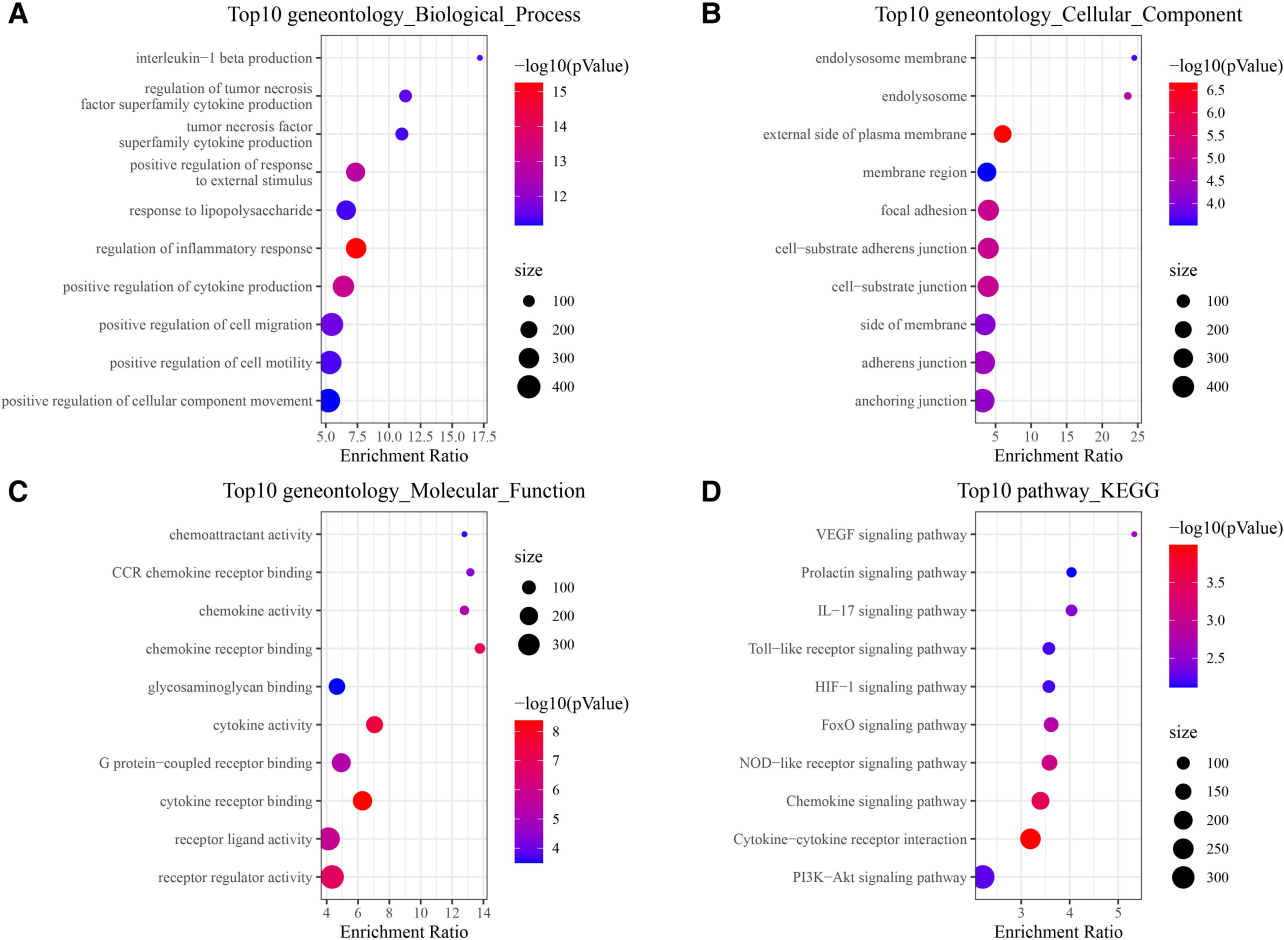
Functional enrichment analysis of 137 target genes. (**A**) BP annotated map. (**B**) CC annotated map. (**C**) MF annotated map. (**D**) KEGG pathways.

For further clarifying multiple mechanisms among the CAD target genes at a system level, GO and KEGG enrichment analysis on the 137 target genes was conducted using WebGestaltR. The top 10 biological process GO entries, molecular function GO entries and KEGG signaling pathways involving these targets were shown in Figures 4A–D. We found that HIF−1 signaling pathway, Chemokine signaling pathway, IL−17 signaling pathway, VEGF signaling pathway, Cytokine−cytokine receptor interaction, PI3K−Akt signaling pathway were enriched.

### Module-based network analysis of potential targets

**Figure 5:**
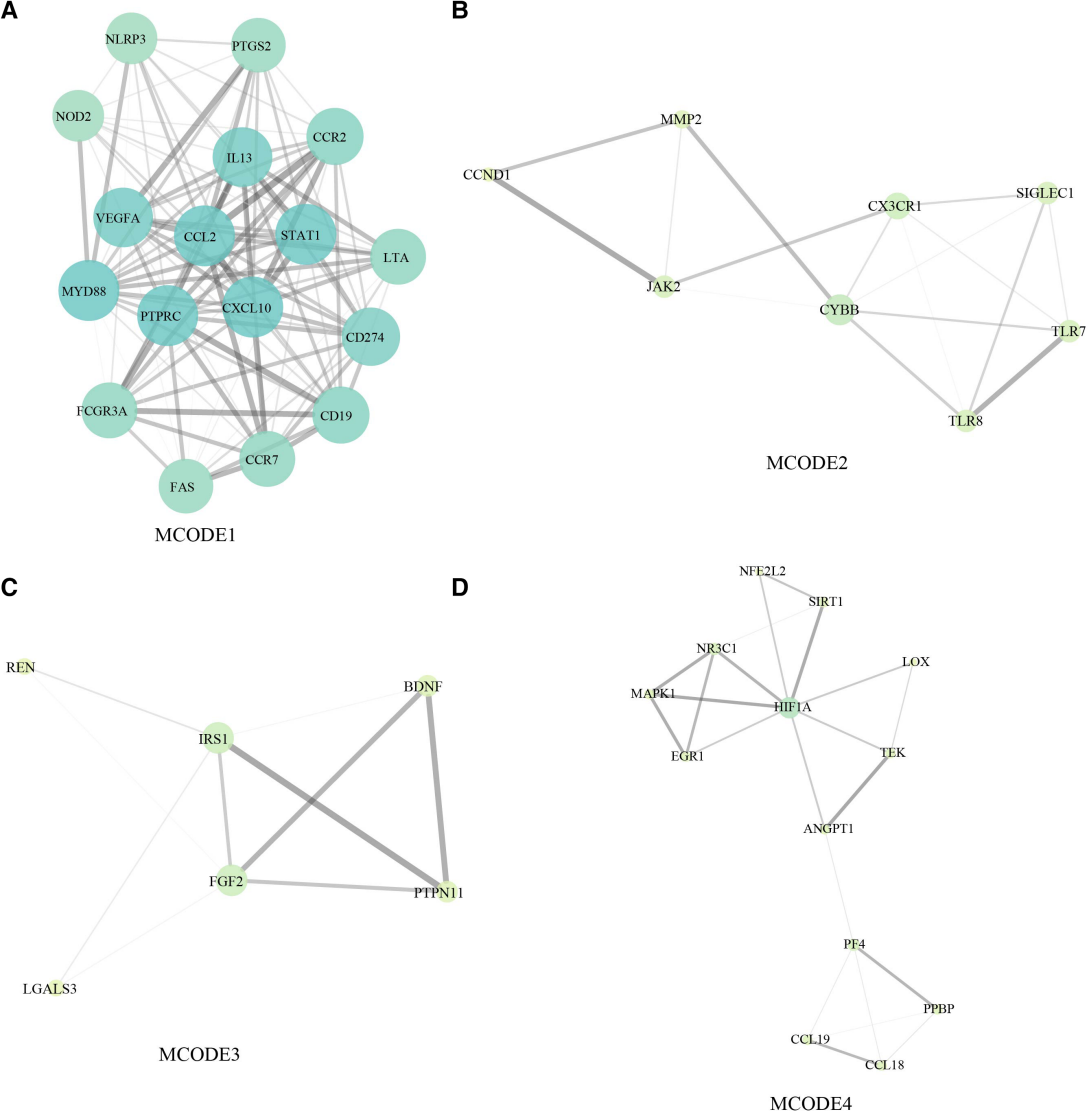
Module-based network of CAD. (**A**) MCODE1. (**B**) MCODE2. (**C**) MCODE3. (**D**) MCODE4.

In the network of potential targets, densely correlated protein groups and the biological functions of each group were found and annotated by MCODE algorithm. From the above 137 targets, we extracted four functional modules (Figures 5A–D).

### Gene-phenotype correlation analysis of CAD

**Figure 6:**
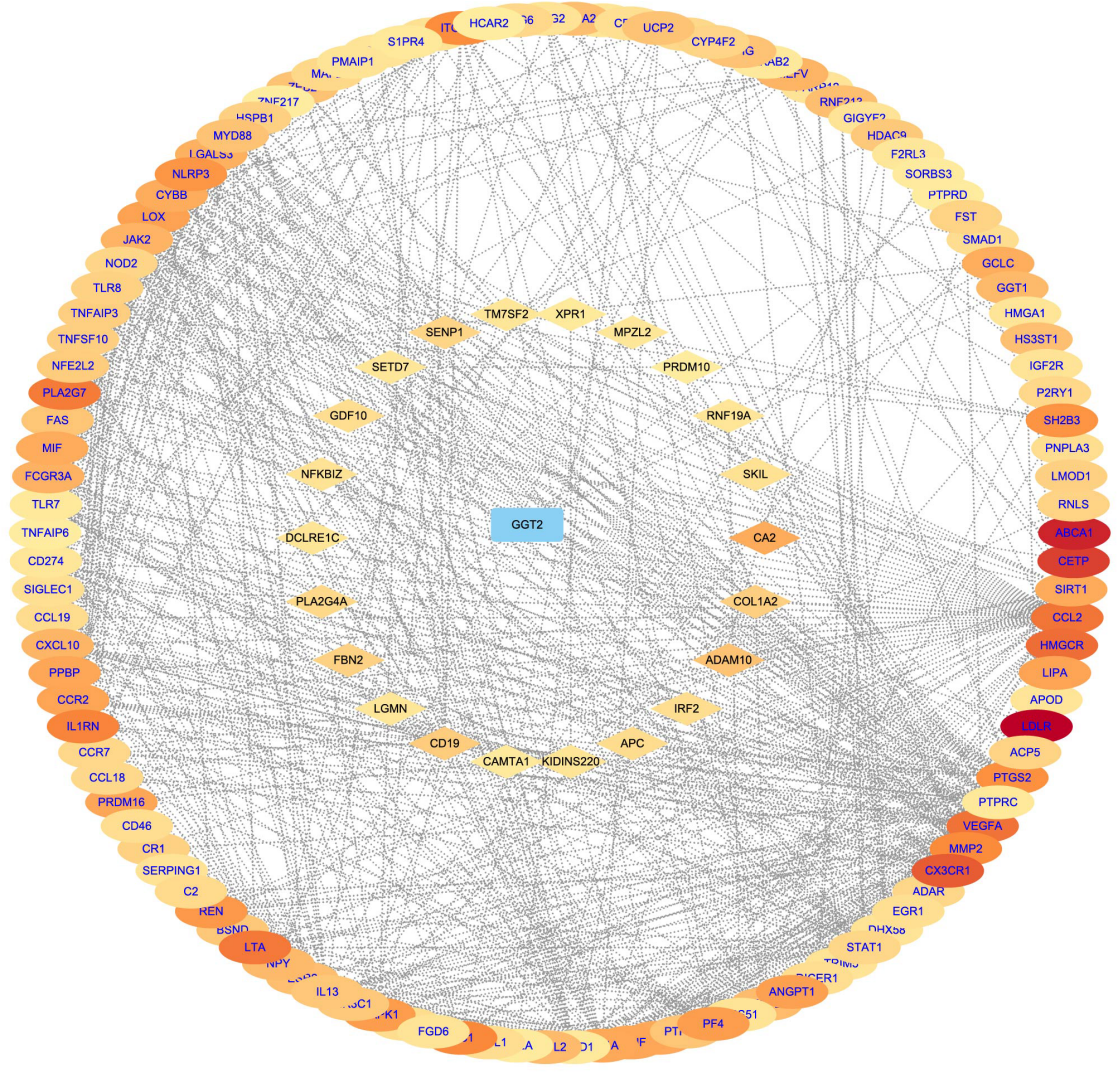
Gene–phenotype correlation analysis of CAD. Intersection computes genes were categorised as directly (oval blue font) or indirectly (diamond black font) associated with the diabetic cataract phenotype. The darker the color, the higher the score.

Direct and indirect genes associated with the CAD phenotype were shown in Figure 5 after correlation study between potential therapeutic targets and the CAD phenotype. The results showed that among the 162 potential targets, 22 were indirectly associated with the CAD phenotype, whereas 161 were directly associated with it (Figure 6). Table 1 displays the top 20 target genes that were indirectly and directly related to CAD phenotype.

**Table 1:** Top 20 targets directly or indirectly associated with the CAD phenotype.

Numbers	Symbol	Description	Type	Direct/Indirect	Score	Average Disease Causing Likelihood
1	LDLR	Low Density Lipoprotein Receptor	Protein	Direct	45.2	58
2	ABCA1	ATP Binding Cassette Subfamily A Member 1	Protein	Direct	36.12	51.7
3	CETP	Cholesteryl Ester Transfer Protein	Protein	Direct	28.75	43.6
4	CX3CR1	C-X3-C Motif Chemokine Receptor 1	Protein	Direct	21.97	15.9
5	HMGCR	3-Hydroxy-3-Methylglutaryl-CoA Reductase	Protein	Direct	17.54	83.1
6	VEGFA	Vascular Endothelial Growth Factor A	Protein	Direct	16.26	64.8
7	CCL2	C-C Motif Chemokine Ligand 2	Protein	Direct	15.59	69.2
8	LTA	Lymphotoxin Alpha	Protein	Direct	14.6	12.1
9	PLA2G7	Phospholipase A2 Group VII	Protein	Direct	13.83	21.8
10	SCN5A	Sodium Voltage-Gated Channel Alpha Subunit 5	Protein	Direct	12.44	21.5
11	MTR	5-Methyltetrahydrofolate-Homocysteine Methyltransferase	Protein	Direct	11.98	44.6
12	IL1RN	Interleukin 1 Receptor Antagonist	Protein	Direct	11.6	79.1
13	IRS1	Insulin Receptor Substrate 1	Protein	Direct	10.63	33.3
14	MMP2	Matrix Metallopeptidase 2	Protein	Direct	9.93	71.8
15	ITGA2B	Integrin Subunit Alpha 2b	Protein	Direct	9.69	50.7
16	PTGS2	Prostaglandin-Endoperoxide Synthase 2	Protein	Direct	9.23	66.5
17	SH2B3	SH2B Adaptor Protein 3	Protein	Direct	8.95	36.8
18	NLRP3	NLR Family Pyrin Domain Containing 3	Protein	Direct	8.74	76.5
19	REN	Renin	Protein	Direct	8.42	75.7
20	MAPK1	Mitogen-Activated Protein Kinase 1	Protein	Direct	8.08	73.8

### The correlation analysis between immune cells and hub genes

**Figure 7:**
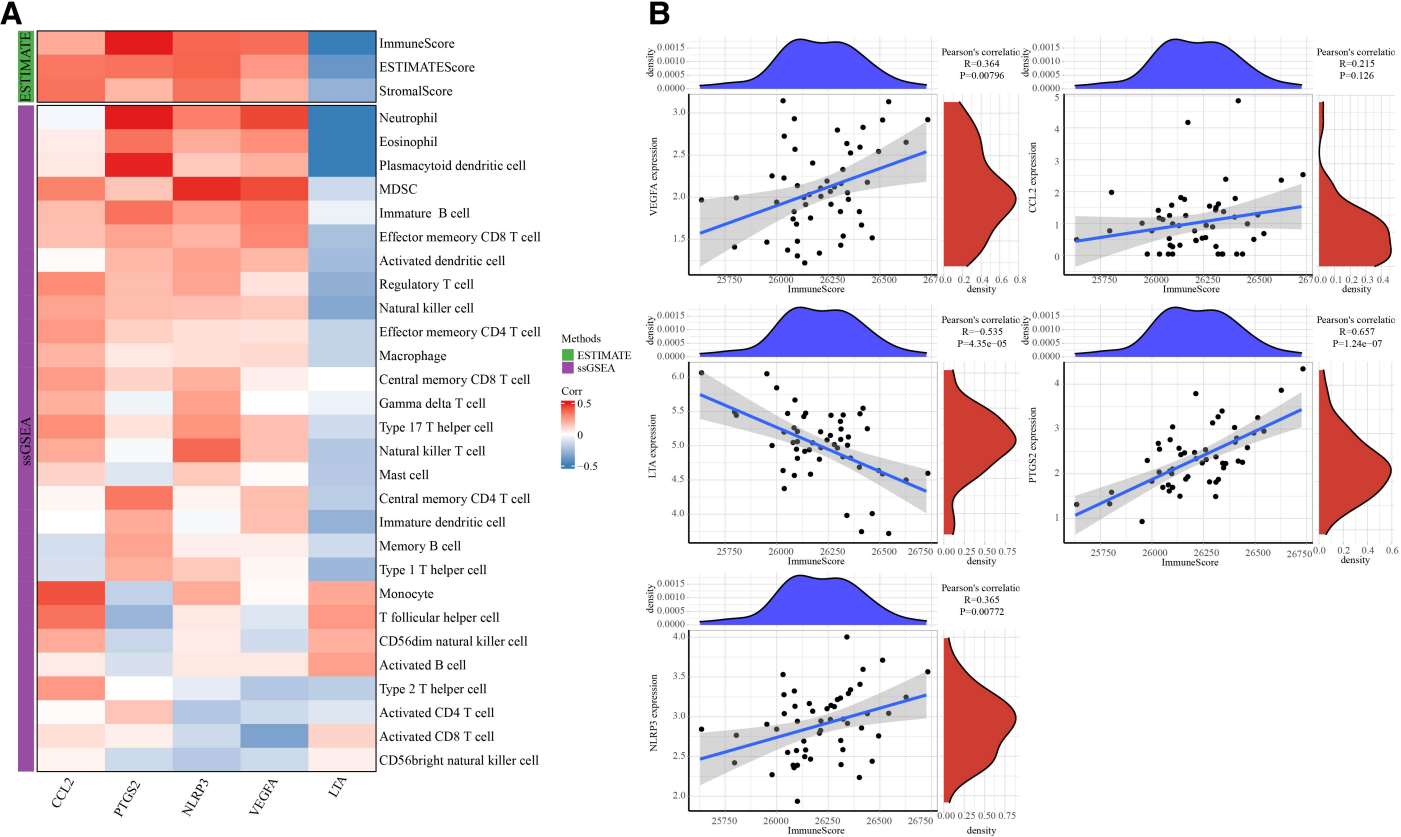
The correlation analysis between 5 hub genes and immunes. (**A**) Heatmap of correlation between immune cells scores and 5 hub genes. (**B**) Correlation between immunescores and 5 hub genes.

5 hub genes were determined by intersection above the top 20 target genes and genes in MCODE1. ESTIMATE analysis and ssGSEA analysis were used to calculate immune cells scores (Figure 7A). LTA expression was negatively correlated with immune score, whereas PTGS2, NLRP3 and VEGFA expressions were positively correlated with immune score (Figure 7B).

### The correlation analysis between mRNAsi and hub genes

**Figure 8:**
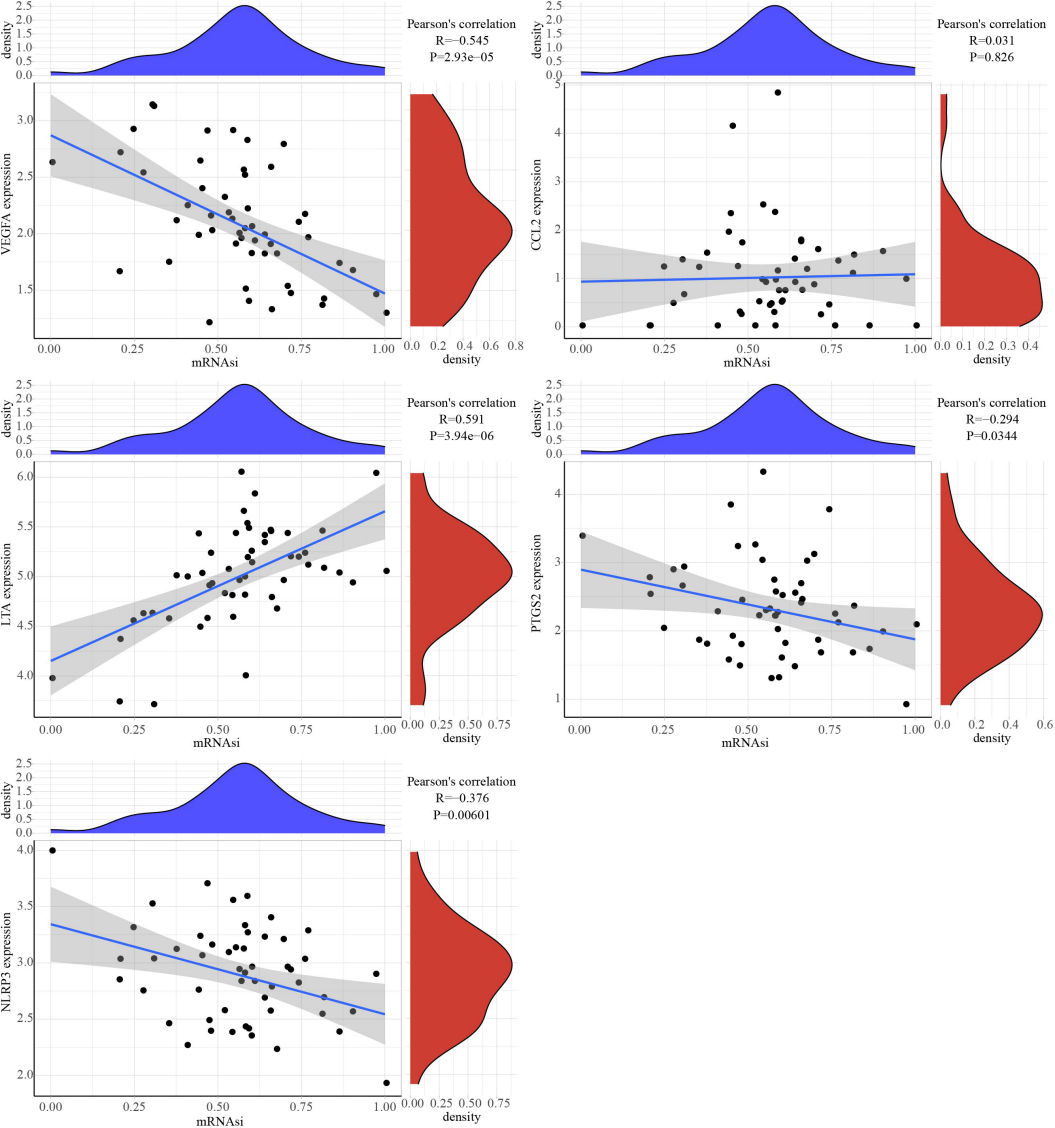
Correlation between mRNAsi and 5 hub genes.

The correlation analysis between 5 hub genes and mRNAsi showed that PTGS2, LTA expression was positively correlated with mRNAsi, while NLRP3 and VEGFA expressions were negatively correlated with mRNAsi (Figure 8).

### Establishment and validation of diagnosis model

**Figure 9:**
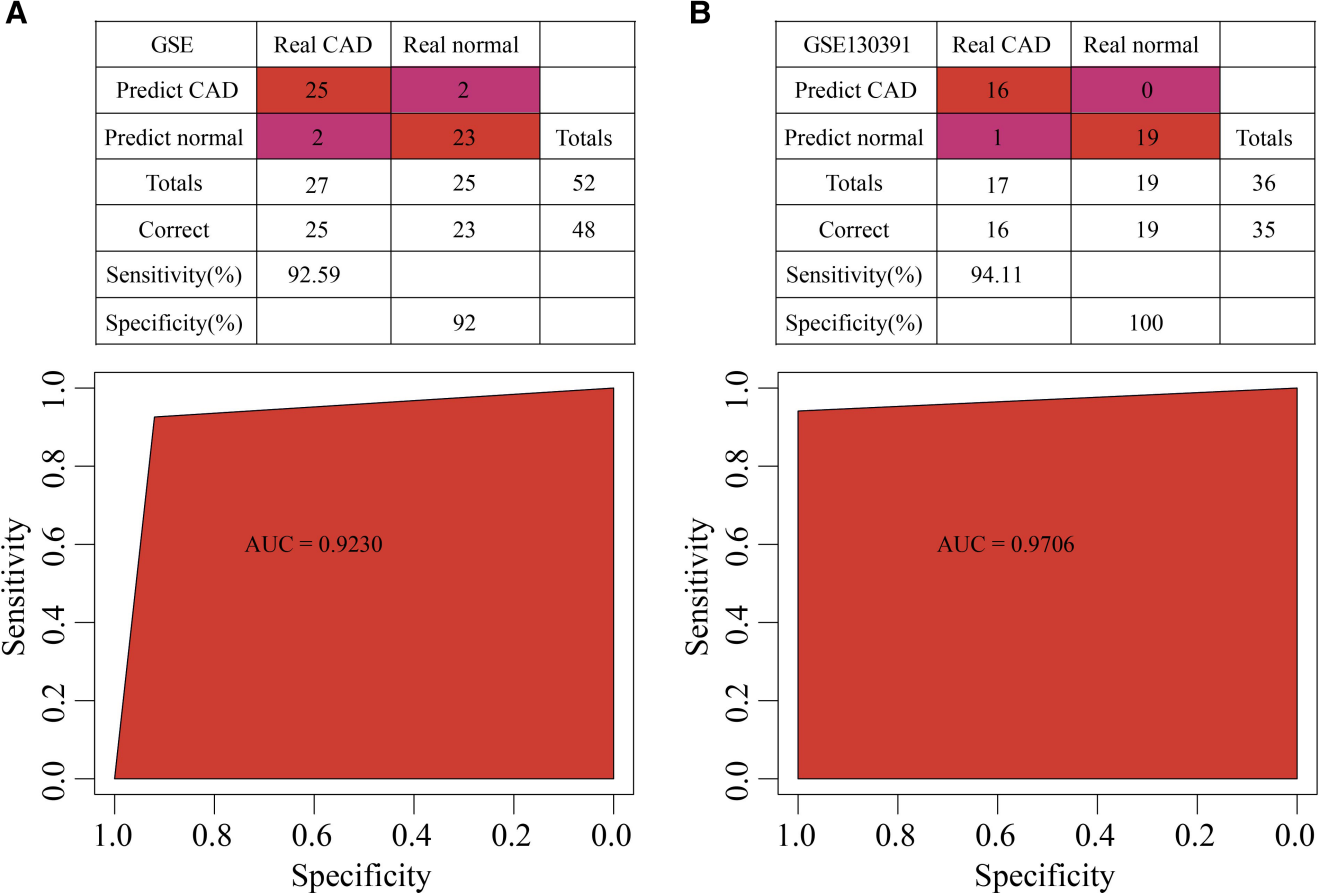
Construction of diagnosis model. (**A**) The classification results and ROC curve of GSE202625 samples in the diagnostic model. (**B**) The classification results and ROC curve of GSE120774 samples in the diagnostic model.

The 5 hub genes were the features in the training dataset (GSE202625 dataset), we obtained their corresponding expression profiles. A SVM classification model was developed. 48 out of 52 samples were correctly classified. The model had a specificity of 92 and a sensitivity of 92.59, and area under the ROC curve (AUC) was 0.92 (Figure 9A). In the validation dataset (GSE120774 dataset), 35 out of 36 samples were correctly classified. The sensitivity of the model was 97.22, the specificity was 100, and the area under the ROC curve (AUC) was 0.9706 (Figure 9B).

## Discussion

Numerous studies have shown that inflammatory factors [tumor necrosis factor alpha, interleukin (IL)-1, and IL-6 levels] are elevated in patients with atherosclerotic heart disease (11, 12). In addition, reduction of the inflammatory response has decelerated the development of atherosclerosis and reduced cardiovascular events (13). Increased white blood cell count has been found to be an independent predictor of AMI death in clinical study (14). Lymphocytopenia is associated with a poor prognosis in various diseases such as stable CAD acute coronary syndrome and heart failure (15–17). In addition, low lymphocyte count is positively associated with the occurrence of cardiovascular events (15). In our study, our identified target genes enriched some Inflammatory factor-related pathways, such as Cytokine−cytokine receptor interaction, Chemokine signaling pathway, IL−17 signaling pathway.

Monocyte activity is associated with clinical indicators of atherosclerosis, heart failure syndrome and CKD (18). Previous studies have shown that CD8 T cells secrete a variety of inflammatory cytokines that exacerbate the inflammatory response and increase atherosclerotic plaque instability (19). Conversely, targeting antigen-presenting cells and modulating the cytotoxic activity of CD8 T cell subsets may inhibit atherosclerosis by attenuating the immune response (19). Other immune cell types, including principal cells (20) and neutrophils (21), also play a key role in the development of cardiovascular disease. Above data suggests that the immune system is very important in the development and progression of CAD. To further evaluate the relationship between immune cells and 5 hub genes in CAD, the ESTIMATE and ssGSEA analysis were used to perform a comprehensive evaluation of immune cells infiltration. The results displayed those 5 hub genes were closely associated immune cells scores.

We next constructed a diagnosis model based on 5 hub genes (CCL2, PTGS2, NLRP3, VEGFA and LTA). Among which, a reporter said that PTGS2 expression was upregulated in advanced stages of atherosclerosis, and positively associated with severity of atherosclerosis (22). As a chemokine, CCL2 expression was significantly elevated in diseased arteries and correlated significantly with the predictive value of atherosclerosis (23). In CAD patients, the NLRP3 gene expression was almost doubled (24). Several studies have reported that VEGFA signaling pathway is involved in CAD (25–27). Previous case-control studies suggested the single nucleotide polymorphisms of LTA gene is associated with CAD and myocardial infarction (28). Those data demonstrated reliability of our diagnosis model.

There are still some shortcomings in this study. The results of this study are only obtained from a public database, and experimental and clinical verification is needed. Secondly, the study is based on a relatively small number of samples, and validation in larger independent datasets would be desirable to confirm the robustness of the diagnostic model. Additionally, the study focuses on gene expression data, but the authors do not provide any information on potential post-transcriptional or post-translational regulation of the target genes, which may impact the validity of the findings. In conclusion, there are significant differences in gene expression during the pathogenesis of CAD, and its pathological process is related to inflammation and other signaling pathways. CCL2, PTGS2, NLRP3, VEGFA and LTA may be key genes in the pathogenesis of CAD.

## Data Availability

The datasets presented in this study can be found in online repositories. The names of the repository/repositories and accession number(s) can be found in the article/**Supplementary Material**.
