# Supplementary material for: Identification of 5 hub genes for diagnosis of coronary artery disease
Source: Front Cardiovasc Med. 2023 Jul 5;10:1086127. doi: 10.3389/fcvm.2023.1086127 (PMC10354867; doi:10.3389/fcvm.2023.1086127)
Supplement: Supplementary file 1 [file Datasheet1.docx]

For the data analyzed in this study please see:

https://www.jianguoyun.com/p/DVqIzdUQ05WMCxj40-IEIAA
